# Editorial: Tobacco disease and biological control

**DOI:** 10.3389/fmicb.2025.1694523

**Published:** 2025-10-03

**Authors:** Digvijay Verma, Zhengkun Zhang, Jinliang Liu

**Affiliations:** ^1^Department of Environmental Microbiology, School of Earth and Environmental Sciences, Babasaheb Bhimrao Ambedkar University, Lucknow, Uttar Pradesh, India; ^2^Jilin Academy of Agricultural Sciences (CAAS), Changchun, China; ^3^College of Plant Sciences, Jilin University, Changchun, China

**Keywords:** smokeless tobacco, Next-Generation Sequencing, tobacco diseases, metagenomics, microbiome

Tobacco-inhabiting microorganisms play a crucial role in determining the quality and yield of tobacco products. Over the past decade, significant advancements have been made in understanding the microbial dynamics of smokeless tobacco. These microbiomes have also been linked to the development of several oral diseases, including oral squamous cell carcinoma (Saxena et al., 2022; Srivastava et al., 2022). Additionally, because of its economic importance, preventing and controlling tobacco-related diseases has been a major concern to improve both yield and quality. Microorganisms play a crucial role in fermentation by enhancing aroma and sensory attributes. Next-Generation Sequencing (NGS) has become vital for uncovering hidden information about uncultivable microorganisms. When combined with meta-transcriptomics, meta-proteomics, and metabolomics, NGS allows for comprehensive analysis of both pathogenic and non-pathogenic microbes. This Research Topic emphasizes the health of economically important tobacco crops. It covers 14 research articles on various facets of microorganisms affecting the health factors, aroma, diseases, and yield of smokeless tobacco.

Physical parameters significantly influence the fermentation process. Zhang et al. observed temperature-dependent improvements to the sensory qualities of cigar tobacco leaves. Furthermore, bacterial diversity in tobacco also showed high levels of *Oceanobacillus* and *Bacillus*, along with *Staphylococcus* and *Corynebacterium*, during increasing temperatures (35 ± 2 °C to 45 ± 2 °C). *Bacillus* spp. play a major role in the tobacco bacterial microbiome (Han et al., 2016; Sajid et al., 2021; Vishwakarma and Verma, 2024). Whole genome sequencing analysis by Wei et al. revealed that *Bacillus halotolerans* NS36 and *Bacillus mycoides* NS75 positively impact the sensory qualities of tobacco. Several reports highlight moisture's vital role in tobacco leaf fermentation. Gao et al. performed NGS-based analysis of cigar tobacco leaves and found that varying water content significantly alters the structure and abundance of bacterial communities. Furthermore, Li et al. conducted an NGS study on cigar tobacco leaves and observed a significant microbial shift due to air curing. Here, *Pseudomonas* and *Sphingomonas* populations were shifted to *Pantoea* from the pre- to post-air-curing stages. This transition resulted in increased pathways related to carbohydrate and amino acid metabolism, leading to the formation of flavor compounds in the middle leaves of tobacco. Wang et al. also performed multi-omics to understand the phyllosphere microbial community. The study concluded that the mid-stage is the most active metabolic phase, so this should be explored to develop biomarkers to assess the fermentation quality.

Fu et al. reported a correlation between fungal diversity and leaf health of tobacco. Healthy and moldy tobacco leaves exhibited significant differences in fungal communities. During analysis, *Aspergillus* was observed as the most harmful mold and notably reduced overall tobacco quality. Moldy tobacco also showed a notable decrease in pH, alkaloids, and flavor compounds, while fatty acid esters increased due to the abundance of *Aspergillus*. Fungi and yeasts influence various plant attributes. Ma et al. observed a significant shift in the rhizosphere bacterial diversity of tobacco due to the oomycete pathogen *Phytophthora nicotianae*. Further analysis revealed the buildup of beneficial microbes in the inter-root zone that form biofilms and activate the plant's immune system against pathogens. Jiang et al. studied the interactions in fungal-bacterial communities that combat Root-Knot Nematode (RKN) caused by *Meloidogyne incognita*. During RKN infection, an abundance of *Microbacterium* spp. and fungi was observed. Such symbiotic bacterial-fungal interactions could be developed as biocontrol agents for other nematodes. Sang et al. found that organic and fatty acids were more prevalent in RKN-infected soil compared to healthy soil, showing positive correlations with *Basidiomycota, Agaricales*, and *Ralstonia*. Whereas, Yang et al. observed that the diversity and count of pathogenic fungi significantly increased in *Fusarium*-infected tobacco roots as compared to the healthy plant. Shotgun-based metagenomic analysis showed a notable decrease in carbon metabolism in diseased tobacco. Mai et al. studied the yeast *Phaffia rhodozyma* for its potential to enhance tobacco aroma via fermentation; this yeast produces the valuable carotenoid astaxanthin, which stimulates sensory molecules in tobacco.

Intercropping has proven to be a fruitful traditional method for improving soil health. This was also reflected in microbial diversity during tobacco-maize relay intercropping. Yang et al. observed higher levels of beneficial microorganisms in intercropped systems compared to monoculture, which assisted in increasing the availability of phosphorus, nitrogen, and potassium in the soil.

The impact of viruses on the overall health of smokeless tobacco plants remains less studied. In a study, Sun et al. expressed the3′ UTR of Tobacco Vein Mottling Virus (TVMV) near Green Fluorescent Protein (GFP) in a tobacco plant. Transcriptome analysis showed that this3′ UTR could activate multiple plant resistance genes, indicating that viral non-coding regions play a pivotal role in plant antiviral defense mechanisms. The study of Wang et al. concludes that copper-zinc superoxide dismutases (Cu/Zn-SOD) play a significant role in the antiviral response in tobacco.

Overall, the Research Topic concludes that microorganisms significantly influence the health of tobacco. Microbes assist fermentation and incorporate flavor-enhancing alkaloids and flavonoids. A healthy microbiome can help to control diseases by stimulating plant defense mechanisms. With advancements in omics technologies, much progress has been made in understanding tobacco microbiology. However, there are lots of basic and applied research on the way, for instance, metabolomics studies are needed to better understand how microbes influence tobacco quality and disease resistance. Additionally, specific active microorganisms must be screened for their functions in tobacco products. Microorganism-pathogen-plant interactions must be verified to support the development of products with more practical and feasible applications.
